# Artificial Intelligence for Spirometry Quality Evaluation: A Systematic Review

**DOI:** 10.3390/bioengineering12121286

**Published:** 2025-11-23

**Authors:** Julia López-Canay, Manuel Casal-Guisande, Cristina Represas-Represas, Jorge Cerqueiro-Pequeño, José-Benito Bouza-Rodríguez, Alberto Comesaña-Campos, Alberto Fernández-Villar

**Affiliations:** 1 NeumoVigo I+i Research Group, Galicia Sur Health Research Institute (IIS Galicia Sur), SERGAS-UVIGO, 36312 Vigo, Spain; julia.lopez@iisgaliciasur.es (J.L.-C.); cristina.represas.represas@sergas.es (C.R.-R.); jose.alberto.fernandez.villar@sergas.es (A.F.-V.); 2 Department of Design in Engineering, University of Vigo, 36208 Vigo, Spain; jcerquei@uvigo.es (J.C.-P.); jbouza@uvigo.es (J.-B.B.-R.); acomesana@uvigo.es (A.C.-C.); 3 Centro de Investigación Biomédica en Red, CIBERES ISCIII, 28029 Madrid, Spain; 4 Pulmonary Department, Hospital Álvaro Cunqueiro, 36312 Vigo, Spain; 5 School of Industrial Engineering, University of Vigo, 36310 Vigo, Spain

**Keywords:** spirometry, quality control, artificial intelligence, intelligent system, deep learning, machine learning, acceptability, error detection

## Abstract

*Background and Objectives*: Spirometry is the most widely used pulmonary function test for diagnosing respiratory diseases. Its progressive incorporation into non-specialized settings, such as primary care, raises challenges for ensuring the reliability of results. In this context, tools based on artificial intelligence (AI) techniques have emerged as promising solutions to support quality control in spirometry. This systematic review aims to synthesize the available evidence on their application in this field. *Methods*: A systematic search was conducted in PubMed and IEEE Xplore to identify peer-reviewed original studies, published between 2014 and June 2025, that applied AI to spirometry quality control. The search and data extraction followed the PRISMA guidelines. *Results*: Six studies met the inclusion criteria. Four analyzed the acceptability and usability of the maneuver, and two focused on detecting errors committed during test performance. The most widely used models were convolutional neural networks, used in four studies, whereas two studies employed other conventional machine learning models. Three models reported area under the ROC curve values higher than 0.88. *Conclusions*: AI-based tools show great potential to assist in spirometry quality control, both in determining acceptability and in detecting errors. However, current studies remain scarce and highly heterogeneous in both objectives and methods. Broader, multicenter research, including validation in non-specialized settings, is required to confirm their clinical utility and facilitate their implementation in clinical practice.

## 1. Introduction

Spirometry is a physiological test that measures the maximum airflow and volume a person can exhale during a forced expiratory maneuver [1]. It is the most used pulmonary function test due to its fundamental role in the diagnosis and monitoring of respiratory diseases such as chronic obstructive pulmonary disease (COPD) and asthma [1]. Moreover, it is a simple, non-invasive, safe, and low-cost technique [2,3], which facilitates its widespread implementation in clinical practice.

Despite its advantages, the main challenge of spirometry lies in ensuring the quality of the maneuvers, as their technical validity can be compromised by several personal and operational factors [4]. Active patient cooperation is essential for a good performance during the test [1], which requires healthcare personnel to provide clear instructions and continuous support throughout the procedure.

The standards of the American Thoracic Society (ATS) and the European Respiratory Society (ERS) [1,4,5] define precise numerical criteria for assessing the acceptability, usability and reproducibility of maneuvers; however, the visual inspection of flow-volume and volume-time curves remains the gold standard for evaluating spirometry quality [6]. This process, although essential, depends on the evaluator’s experience and shows considerable inter-observer variability, which may lead to technically invalid maneuvers being classified as acceptable maneuvers. Such errors compromise the interpretation of results and may lead to incorrect diagnoses or inadequate patient follow-up.

The training and experience of technical staff are therefore key factors in ensuring the reliability of spirometry results [1]. However, not all professionals have the specific training required. According to Hueto et al. [7], only 64% of the technical staff performing spirometry in primary care have received specialized training, which increases the risk of errors in quality control assessment and, consequently, diagnostic errors [7,8].

In recent years, the demand and importance of spirometry have increased significantly, driven by the high prevalence of chronic respiratory diseases, population aging, and the impact of environmental pollution on respiratory health [9]. To address this need, initiatives have been promoted to ensure the performance of high-quality spirometry across all healthcare settings [2]. The aim is to promote early diagnosis and reduce the high rate of underdiagnosis that persists in some respiratory diseases such as COPD [10]. However, the availability of trained personnel has not grown at the same pace as the expansion of spirometry testing, leading to a shortage of expert supervision and an increased risk of technical or interpretative errors in quality control assessment.

In this context, Artificial Intelligence (AI) emerges as a promising tool to automate the verification of the technical quality of spirometric maneuvers, reducing inter-observer variability and providing immediate feedback during test performance.

AI has proven its usefulness in multiple healthcare areas, where it is applied to optimize clinical decision-making and improve the diagnosis, monitoring, and treatment of various diseases [11,12,13,14,15]. In this context and given the challenges associated with quality control in spirometry, advances in the fields of machine learning (ML) and deep learning (DL) have driven the development of intelligent systems capable of detecting errors, classifying maneuvers, and automatically assessing their acceptability and usability. These AI-based tools have shown promising results in clinical practice; for instance, in the study conducted by Topole et al., an AI-based software achieved a 73% agreement with gold-standard labels in the evaluation of spirometry quality [16].

This systematic review aims to synthesize and analyze the most recent evidence on the development of AI-based tools applied to spirometry quality control, identify the main trends, limitations, and knowledge gaps in the literature, and propose future research directions to help optimize their clinical implementation.

## 2. Materials and Methods

### 2.1. Study Design

A systematic literature review was conducted with the aim of providing a summary of the use of AI-based tools applied to spirometry quality control, identify trends and limitations, and provide a framework to support future research.

This study was carried out following the guidelines of the Preferred Reporting Items for Systematic Reviews and Meta-Analyses (PRISMA) [17].

### 2.2. Inclusion and Exclusion Criteria

Studies published between 2014 and 2025, in Spanish or English, with full text availability, that described the development or validation of AI-based tools applied to spirometry quality control using data from clinical spirometry tests performed in humans were included.

Studies were excluded if they did not explicitly apply AI techniques, did not detail the development or architecture of the system, were not peer-reviewed original articles, or did not evaluate model performance using at least one of the following metrics: area under the ROC curve (AUC), sensitivity, or specificity.

### 2.3. Literature Search Strategy

A comprehensive search was conducted in the PubMed and IEEE Xplore databases to identify studies that used AI techniques in the development of tools applied to spirometry quality control. The search strategy included the following keywords: (“Expert System” OR “Intelligent System” OR “Artificial Intelligence” OR “AI” OR “Machine Learning” OR “Deep Learning” OR “Neural Network” OR “Decision Support System” OR “Smart System”) AND (“Spirometry” OR “Pulmonary Function Test” OR “Lung Function Test” OR “PFT”) AND (“Error” OR “Incorrect” OR “Invalid” OR “Acceptability” OR “Quality Assurance” OR “Quality Control” OR “Quality Assessment” OR “Usability” OR “Reproducibility”). The search was limited to articles published between January 2014 and 30 June 2025. Additionally, articles previously known by the authors and the reference lists of the retrieved studies were thoroughly reviewed to identify potential additional publications.

The results were manually screened to remove duplicates. Study selection was conducted in two phases: first, by reading titles and abstracts, and subsequently by reviewing the full text. This process was carried out by two independent reviewers (J.L.-C. and M.C.-G.). In case of discrepancies, a third reviewer (A.F.-V.) was consulted.

### 2.4. Synthesis of the Results

Data extracted from the selected studies were organized into three summary tables presenting their main methodological characteristics and the most relevant results. The aspects analyzed included the study location, data source and origin, sample size, patient age range, clinical setting where data were collected, spirometry device used, labeling process, model employed, input features for the intelligent system, classes defined for spirometry classification, validation strategy, and performance metrics including AUC, sensitivity, and specificity.

A meta-analysis was not feasible due to the heterogeneity observed among the studies in terms of design, methodology, classification objectives, input features, and validation strategies employed.

## 3. Results

### 3.1. Study Selection

A total of 60 articles were identified in PubMed and 41 in IEEE Xplore, in addition to 4 more articles retrieved manually. After removing duplicates, 100 references remained.

**Figure 1:**
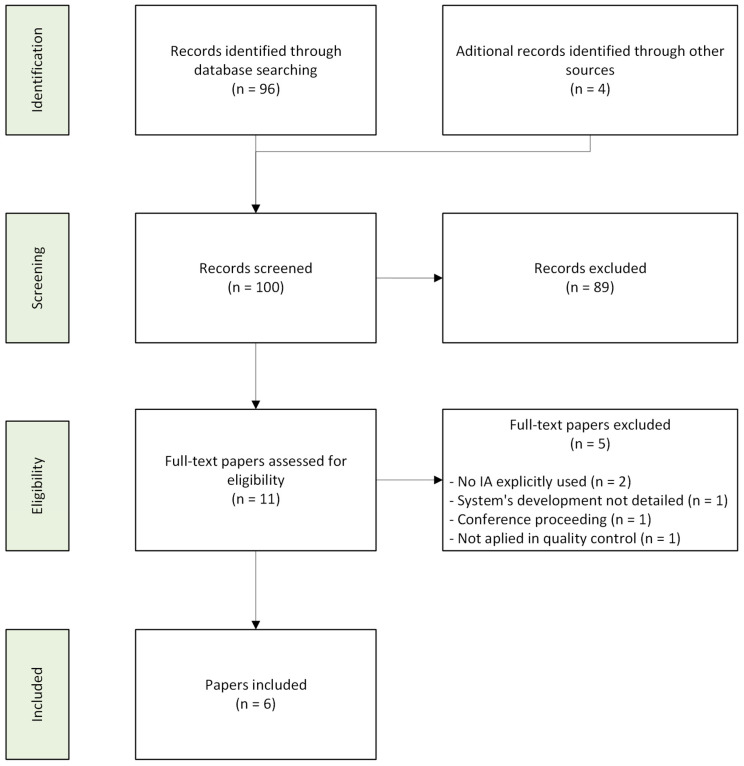
PRISMA flow diagram of the study selection process.

Of these articles, 11 were selected for full-text review after screening titles and abstracts, of which 6 were included in the systematic review. The selection process is illustrated in Figure 1, following the PRISMA methodology [17].

### 3.2. Characteristics of the Included Studies

The included studies were published between 2020 and 2024 and were conducted in Spain, Poland, China, Belgium, the United States of America (USA), and India. All of them developed AI-based tools applied to spirometry quality control. The descriptive characteristics of the studies are presented in Table 1.

The source and type of data used for training and validating the intelligent system varied across studies. Two studies used data from the NHANES database (USA) [18,19], and one of these combined these data with the AioCare database (Poland) [19], which was also used independently in another study [9]. The rest of the studies employed proprietary databases [20,21,22]. The sample sizes also varied substantially, ranging from 900 to 36,873. This variation is closely tied to the source of the data; in general, studies that relied on proprietary databases tended to have smaller sample sizes [20,22].

There is also considerable heterogeneity in patient age ranges. While Velickovski et al. [20] used data from adults over 20 years of age, Das et al. [18] and Solinski et al. [19] included patients aged 6–79 years, and Walag et al. [9] included both children aged 9–15 years and individuals from general population.

Two of the studies used data from spirometry tests performed in primary care [9,20], with one of them also incorporating tests performed in primary schools [9]. Two other studies relied on spirometry tests conducted in hospital settings [21,22], while the remaining two did not specify the clinical setting [18,19]. In two studies, testing was conducted using an AioCare portable spirometer (HealthUp, Poland) [9,19], while the remaining studies either employed different devices [20,21,22] or did not specify the equipment used [18].

The labeling process was described in five of the six studies [9,18,19,20,21]. Four of these reported following the ATS/ERS [1,4,5] criteria to classify tests based on quality [9,18,19,21]. Nevertheless, the labeling procedures varied across the studies.

**Table 1:** Descriptive characteristics of the studies.

Author	Country	Dataset Source and Origin	Sample Size	Patient Age Range	Clinical Setting	Device	Labeling Process
Velickovski et al. [20]	Spain	Basque Country, Spain	900	>20 years	Primary care centers participating in forced spirometry training	Sibel 120 (SIBELGroup, Barcelona, Spain) operating at 100 Hz	Spirograms were randomly selected and independently evaluated by three clinical experts from the Lung Function Unit at the Hospital Clinic of Barcelona. Each expert assessed 600 spirograms, labeling them as acceptable if at least two experts agreed on acceptability, and rejected if at least two agreed on rejection. Spirograms for which there was no consensus between the experts were excluded from the dataset.
Walag et al. [9]	Poland	AioCare database (Poland)	1998	Children aged 9–15 and general population	Primary care and primary schools	AioCare portable spirometer (HealthUp, Warsaw, Poland)	Spirograms were visually inspected by experienced pulmonologists and labeled according to ATS/ERS 2019 criteria, assessing the acceptability and usability of FEV_1_ and FVC separately. A spirogram was labeled as confirmed if it was acceptable or usable, and rejected otherwise. Spirograms without the consensus of at least three experts were excluded.
Wang et al. [21]	China	Pulmonary function tests databases of the First Affiliated Hospital of Guangzhou Medical University	16,502	-	Hospital	MasterScreen-Pneumo (Jaeger, Hanover, Germany)	Spirograms were visually inspected and labeled according to ATS/ERS 2005 guidelines and Chines Thoracic Society 2014 guidelines by four pulmonologists. Any uncertainties were resolved by an independent expert.
Das et al. [18]	Belgium	NHANES 2011–2012 database	36,873	6–79 years	-	-	Spirograms were reviewed according to ATS/ERS 2005 criteria and classified into four groups: A: acceptable quality, B: large time to peak flow or non-repeatable peak flow; C: <6 s exhalation or no plateau; D: cough or back extrapolated volume. Curves labeled A were considered acceptable, and curves labeled A, B, or C were considered usable. Curves labeled D were excluded.
Solinski et al. [19]	Poland	NHANES 2007–2012 database, AioCare database and steady-flow signals	19,832	NHANES: 6–79 years AioCare: 7–80 years	-	AioCare portable spirometer (HealthUp, Poland) and Series 1120 Flow Volume Simulator by Hans Rudolph, Inc. (Shawnee, KS, USA)	Raw signals of the spirometry curves are divided in 4 groups: A: acceptable quality; B: large time to peak flow or non-repeatable peak flow; C: <6 s exhalation or no plateau; D: cough and back extrapolated volume The cough containing curves were extracted from the D group by 4 experts. Finally, two groups were created: ATS-acceptable and other error curves and cough curves.
Bonthada et al. [22]	India	JSS Hospital, Mysore, India	1600	-	Hospital	Spirobank G spirometer (Morgan Scientific Inc., Haverhill, MA, USA)	-

### 3.3. Main Results

Two main types of AI-based tools for spirometry quality control were identified: on one hand, those aimed at determining the acceptability and usability of the tests and, on the other hand, those focused on detecting errors during test performance.

A maneuver is considered acceptable if it meets all ATS/ERS criteria [1,4,5], which vary depending on the parameter evaluated (FEV_1_ or FVC). A maneuver is considered usable if it does not meet all the acceptability criteria but remains clinically useful. The usability criteria also vary on whether FEV_1_ or FVC is assessed.

Across studies, the types of errors evaluated were heterogeneous. Reported categories included extra breaths, high back-extrapolated volume, early termination, submaximal effort, and cough, but definitions and labeling methods varied, limiting direct comparability.

No studies were found that evaluated both aspects simultaneously.

#### 3.3.1. Systems Oriented Towards the Determination of Acceptability and Usability

Four studies focused on evaluating the acceptability or usability of spirometry [9,18,20,21]. Table 2 shows a summary of the methodological characteristics of these articles.

In all cases, binary classifiers were implemented, although three studies developed multiple independent classifiers for different parameters [9,18,21]. Most of the studies used convolutional neural networks (CNNs): two exclusively [9,18] and one in combination with a rule-based module [21]. Only one study employed traditional ML algorithms, with the Random Forest-based model achieving the best performance [20].

The type of input data varied among studies, showing considerable heterogeneity. Among those that used CNNs, one processed flow-time signals [9], another used 32 × 32-pixel images of the flow-volume curves [18] and the last used 600 × 1200-pixel images of the flow-volume and volume-time curves [21]. In contrast, Velickovski et al. [20] employed a normalized vector with two spirometric parameters, the backward extrapolated volume (BEV) and the forced expiratory time (FET), along with seven coefficients derived from the polynomial fitting of the flow-time curve.

Regarding the objectives, all models evaluated the acceptability of spirometry, and three also included usability. In two studies, both criteria were analyzed separately for the FEV_1_ and FVC parameters. For example, Walag et al. [9] determined whether FEV_1_ and FVC met the acceptability or usability criteria, while Wang et al. [21] implemented four independent classifiers for each combination of parameter and criterion. In the study by Das et al. [18], acceptability was evaluated globally, whereas usability was analyzed only for FEV_1_ using a specific classifier.

All studies validated their models using an independent internal test set. Three reported AUC values ranging from 0.88 to 0.98 [9,18,20]. Reported sensitivities ranged from 60.0% to 99.5%, while specificities ranged from 85.0% to 96.0%. Additionally, Wang et al. [21] assessed the clinical impact of the intelligent system developed in their study, demonstrating improvements in the proportion of good-quality tests for FEV_1_ and FVC by approximately 21% and 36%, respectively.

**Table 2:** Summary of the studies oriented towards the determination of acceptability and usability.

Author	Model	Features	Classes	Validation	AUC	Sensitivity	Specificity
Velickovski et al. [20]	5 ML models: Naïve Bayes, kNN, logistic regression, SVM, Random Forest	Normalized vector: 2 spirometric parameters (BEV, FET) + 7 coefficients derived from the polynomial fitting of the flow-time curve	(1) acceptable/(2) rejected	422 (70%) training with 10-fold cross-validation; 181 (30%) test	0.88	60%	91%
Walag et al. [9]	CNN	Flow-time signals	FEV_1_ (1) confirmed (acceptable or usable)/(2) rejected	1561 (80%) train; 391 (20%) test	0.95	93.1%	90.0%
			FVC (1) confirmed (acceptable o usable)/(2) rejected	1569 (80%) train; 393 (20%) test	0.98	95.6%	88.3%
Wang et al. [21]	Rule based module + object detection module (Faster R-CNN with ResNet50)	Numeric parameters (pulmonary function parameters, data from flow-volume and volume-time curves) + 600 × 1200 images of flow-volume and volume-time curves	FEV_1_ (1) acceptable/(2) not acceptable	14,124 (90%) train; 1569 (10%) test	-	97.8%	92.4%
			FEV_1_ (1) usable/(2) not usable		-	99.4%	85.4%
			FVC (1) acceptable/(2) not acceptable		-	97.5%	89.6%
			FVC (1) usable/(2) not usable		-	99.5%	89.0%
Das et al. [18]	CNN	32 × 32 images of the flow-volume curve	(1) acceptable/(2) not acceptable	29,452 (80%) train; 3683 (10%) validation; 710 (10%) test	0.93	90.0%	85.0%
			FEV_1_ (1) usable/(2) not usable		0.98	92.0%	96.0%

#### 3.3.2. Systems Oriented Towards Error Detection

Two studies focusing on the detection of errors during spirometry performance were identified [19,22]. Both employed binary classifiers to distinguish between the presence and absence of performance errors, but the types of errors assessed differed between studies.

For example, Bonthada et al. [22] developed a binary classifier to detect the presence of performance errors, including extra breaths, high back-extrapolated volume, early termination, and submaximal effort. In addition, they implemented a multiclass classifier capable of distinguishing between these four error types individually. In contrast, Solinski et al. [19] evaluated only a single type of performance error, specifically the presence or absence of cough. Table 3 presents a summary of these studies.

The applied models differed across studies. Bonthada et al. [22] implemented two independent CNNs, whose outputs were integrated through a multilayer perceptron (MLP). On the other hand, Solinski et al. [19] compared different ML algorithms, including logistic regression, feed-forward neural networks, support vector machines (SVMs), and Random Forest, with the model based on artificial neural networks achieving the best performance.

The input variables also varied depending on the approach. Bonthada et al. [22] used 128 × 128-pixel images of the flow-volume and volume-time curves. In contrast, Solinski et al. [19] employed numerical parameters derived from the curves, such as the number of local maxima, horizontal crossings at 15%, 25%, 50%, and 75% of the peak expiratory flow (PEF), as well as local maxima after PEF with a right slope greater than 0.25 L/s.

None of the studies reported AUC values. However, both reported sensitivity and specificity values achieved during validation on an internal test set, showing good results.

**Table 3:** Summary of studies on error detection.

Author	Model	Features	Classes	Validation	AUC	Sensitivity	Specificity
Bonthada et al. [22]	2 independent CNNs + MLP	128 × 128 × 1 images of the flow-volume and volume-time curves	(1) Presence of performance errors/(2) Absence of performance errors	1440 (90%) training 5-fold stratified cross-validation; 160 (10%) test	-	98.0%	87.0%
			(1) Extra breaths/(2) High extrapolated volume/(3) Early termination/(4) Submaximal effort			-	-
Solinski et al. [19]	4 models: logistic regression, feed-forward artificial neural network, SVM and Random Forest	Number of local maxima (>0.05 s), horizontal crossings at 15%, 25%, 50%, 75% of PEF, local maxima after PEF with a right slope greater than 0.25 L/s	(1) Presence of cough/(1) Absence of cough	11,719 (59%) train; 6470 (33%) 5-fold cross validation, 1643 (8%) test	-	86.0%	91.0%

## 4. Discussion

### 4.1. Main Findings

In this systematic review, six original studies were identified that used AI tools for quality control in spirometry. These studies were grouped into two main categories according to their objective: on one hand, four studies used AI tools to assess the acceptability and usability of spirometry tests [9,18,20,21]; on the other hand, two studies applied AI to detect patient errors during test performance [19,22].

Most of the studies employed DL techniques; specifically, four used CNNs [22], while only one relied on feed-forward neural networks [19], which proved to be the best-performing model compared with other ML approaches. On the other hand, only two studies applied conventional ML techniques [19,20]; however, only one of them reported an optimal performance [20], with the Random Forest algorithm standing out as the method that achieved the best results.

The performance of the classifiers was evaluated using the AUC, reported in three of the studies, as well as sensitivity and specificity values, which were reported in all studies. The AUC values ranged from 0.88 to 0.98. In studies that used AI to determine the acceptability or usability of spirometry tests, sensitivity ranged from 60.0% to 99.5%, and specificity from 85.0% to 96.0%. On the other hand, in the studies focused on error detection, sensitivity ranged from 86.0% to 98.0%, while specificity ranged from 87.0% to 91.0%.

### 4.2. Limitations of the Included Studies

The included studies present several methodological and design limitations. First, there is notable heterogeneity in terms of objectives, types of data used, applied models, and validation strategies, which hinders direct comparison between the different systems and their results. Furthermore, not all studies reported AUC values, making it difficult to assess classifier performance in those cases.

Secondly, there are limitations related to the cohorts used. The databases employed varied greatly in size, ranging from 900 to 36,873 samples, and several databases were used in more than one study. Specifically, the NHANES database was used in two studies, as well as the AioCare database. Moreover, only two studies explicitly used data from spirometry tests conducted in primary care settings, which limits the practical applicability of the results in that context. This setting is particularly relevant, as automation of quality control could have a great clinical impact by helping to address the shortage of specialized personnel.

It is also worth noting the heterogeneity of the equipment used to perform the tests. Only two studies employed the same device, resulting in datasets with very diverse characteristics across studies, limiting the comparability of the results.

Finally, quality control in spirometry fundamentally relies on the visual inspection of spirometric curves. This is a subjective task that depends on the training and experience of the technician supervising the test. Despite this, only three of the reported studies used images of the spirometric curves as input features for their systems.

### 4.3. Clinical Implications

Quality control in spirometry is a complex task that requires professionals with specific training [1]. The growing prevalence of chronic respiratory diseases such as COPD, population aging, and the impact of environmental pollution have driven the expansion of this test into non-specialized settings, such as primary care [2,9]. However, the availability of spirometers and the increased demand for testing have not always been accompanied by adequate training of the professionals responsible, which increases the risk of technical and diagnostic errors.

In this context, AI emerges as a promising alternative for the automation of quality control in spirometry. The included studies suggest that AI can:

- Assist quality control by enabling the acquisition of high-quality spirometry tests that reflect the expertise of specialized personnel in non-specialized settings, such as primary care.
- Reduce variability in the interpretation of spirometry quality.
- Reduce errors in the diagnosis of respiratory diseases.
- Detect specific errors made by the patient during the performance of the spirometry test.

These applications help ensure the performance of high-quality spirometry in non-specialized settings, facilitating the early diagnosis of respiratory diseases such as asthma and COPD.

#### 4.3.1. Practical Implementation and Workflow Integration

The AI-based tools identified in this review can be incorporated into daily clinical practice during spirometry quality assessment to enhance the accuracy, consistency, and overall reliability of spirometry testing:

- Determination of spirometry acceptability and usability: these systems could be directly integrated into spirometers. In this way, once the patient has performed the maneuver, the intelligent system can automatically trigger alerts for invalid attempts and provide immediate feedback to the operator regarding the need to repeat the test. Additionally, unacceptable maneuvers would be recorded and classified as invalid within the system, reducing the likelihood of diagnostic errors.
- Detection of errors made by the patient during test performance: these systems can be integrated directly into the spirometer and allow immediate identification of whether the patient has made an error during the maneuver. This approach offers a dual benefit: on one hand, it indicates that the test is unacceptable, and on the other, it specifies the type of error committed. In this way, feedback is provided to both the technician and the patient, facilitating error correction and increasing the likelihood of obtaining an acceptable maneuver upon repetition.

In both cases, the implementation of the tool in clinical practice requires multicenter validation, monitoring of the model’s performance, periodic recalibration, and transparency in the interpretation of results, ensuring confidence and safety in its use in clinical practice.

#### 4.3.2. Adoption Barriers and Enablers

Among the main barriers to adoption are limitations in data availability, including small sample sizes, low population heterogeneity, and a lack of prospective studies. For example, Das et al. [18] used the NHANES 2011–2012 database, which predominantly includes healthy individuals, while Velickovski et al. [20] relied on data collected using a single type of spirometer. This limitation highlights a major challenge for implementing these systems in clinical practice, as variations among spirometer models may not only restrict the generalizability of the intelligent system but also lead to potential interoperability issues. Additionally, the absence of feedback for failed maneuvers constitutes a further limitation to their adoption in clinical practice. This challenge reflects a broader issue with DL-based systems, where interpretability remains a critical factor for clinical acceptance. Other barriers include the lack of external validation and inconsistencies in protocols for performing maneuvers.

In contrast, facilitating factors include access to multicenter databases, such as NHANES and AioCare; the existence of well-defined standards for conducting the tests; and the direct integration of intelligent systems into spirometers, which allows for immediate feedback during the execution of maneuvers and reduces errors. Additionally, these tools contribute to the automation of spirometry quality control in non-specialized settings, such as primary care, and are compatible with existing clinical workflows, which facilitates their implementation in routine practice.

### 4.4. Future Opportunities and Need for Further Analysis

Currently, the use of AI tools for quality control of spirometry remains limited, representing an opportunity to develop systems capable of ensuring the performance of high-quality tests, even in non-specialized settings. Future research could focus on:

- Advanced analysis of spirometric curves using DL: implementing neural networks to support visual inspection, reducing the subjectivity inherent in manual validation.
- Development of integrative representations: combining images of spirometric curves with numerical parameters included in the report, aiming to build more comprehensive and robust classifiers.
- Multicenter and prospective validation: evaluating the models in diverse clinical settings to ensure their applicability, reliability, and scalability.
- Integration with clinical systems and real-time feedback: enabling intelligent systems to provide immediate alerts to the operator and connect with electronic records to improve traceability and the quality of test control.

### 4.5. Limitations of This Review

This systematic review has several limitations that should be considered when interpreting its results. First, the small number of studies identified, along with the heterogeneity in their designs, methodological approaches, and reported outcomes, prevented the conduct of a meta-analysis. Although strict inclusion criteria were applied, it is possible that some relevant articles were not captured, particularly those in which methodological details were not explicitly described in the title or abstract.

Additionally, the search was limited to publications in English and Spanish, which may introduce a language bias and limit the representativeness of the findings. Although the search strategy was applied to widely used databases such as PubMed and IEEE Xplore, which reflect the interdisciplinary nature of the theme, the total number of retrieved publications was limited, which could restrict the generalizability of the conclusions.

Also, the included studies reported different metrics, and not all provided AUC values or evaluated clinical impact. This heterogeneity limits comparability between studies, potentially introducing optimistic bias due to limited validation.

Finally, given the exploratory scope and the marked heterogeneity among the included studies, a structured quality-assessment tool (e.g., PROBAST) was not applied. Although discrepancies during screening and data extraction were minimal and resolved by consensus, this still represents a limitation of the review.

## 5. Conclusions

This systematic review shows that AI has been applied to the quality control of spirometry, mainly through DL techniques based on convolutional neural networks. The studies analyzed report promising results, with high values of AUC, sensitivity, and specificity. However, research in this field is still limited and exhibits considerable heterogeneity in both objectives and methodologies.

Future research should focus on developing more comprehensive intelligent systems capable of simultaneously evaluating the acceptability of maneuvers and detecting user errors. This would enable immediate feedback and promote the efficient conduct of high-quality spirometry. Likewise, it is essential to encourage studies that complement traditional visual inspection and facilitate the optimal integration of these tools into clinical workflows.

Finally, it is a priority to guide these advances toward non-specialized settings, such as primary care, where AI-based systems can have a significant impact by reducing diagnostic errors, improving test accuracy, and increasing efficiency.

## Data Availability

The original contributions presented in this study are included in the article.
